# Detailed Componential Characterization of Extractable Species with Organic Solvents from Wheat Straw

**DOI:** 10.1155/2017/7305682

**Published:** 2017-11-01

**Authors:** Yong-Chao Lu, Yao Lu, Zhao-Lin Lu, Xian-Yong Wei

**Affiliations:** ^1^School of Basic Education Sciences, Xuzhou Medical University, Xuzhou 221004, China; ^2^Advanced Analysis & Computation Center, China University of Mining & Technology, Xuzhou 221116, China; ^3^Key Laboratory of Coal Processing and Efficient Utilization, Ministry of Education, China University of Mining & Technology, Xuzhou 221116, China

## Abstract

Componential analysis of extractives is important for better understanding the structure and utilization of biomass. In this investigation, wheat straw (WS) was extracted with petroleum ether (PE) and carbon disulfide (CS_2_) sequentially, to afford extractable fractions EF_PE_ and EF_CS2_, respectively. Detailed componential analyses of EF_PE_ and EF_CS2_ were carried out with Fourier transform infrared (FTIR) spectroscopy, gas chromatography/mass spectrometry (GC/MS), X-ray photoelectron spectroscopy (XPS), transmission electron microscopy (TEM), energy dispersive spectrometry (EDS), and electron probe microanalysis (EPMA). Total extractives were quantified 4.96% by weight compared to the initial WS sample. FTIR and GC/MS analyses results showed that PE was effective for the extraction of ketones and waxes derived compounds; meanwhile CS_2_ preferred ketones and other species with higher degrees of unsaturation. Steroids were enriched into EF_PE_ and EF_CS2_ with considerable high relative contents, namely, 64.52% and 79.58%, respectively. XPS analysis showed that most of the C atoms in extractives were contained in the structures of C-C, C-COOR, and C-O. TEM-EDS and EPMA analyses were used to detect trace amount elements, such as Al, Si, P, S, Cl, and Ca atoms. Detailed characterization of extractable species from WS can provide more information on elucidation of extractives in biomass.

## 1. Introduction

Biomass has been considered to be a promising feedstock for fuels and chemicals production to substitute fossil resources [1, 2]. The main components of lignocellulosic biomass are cellulose, hemicellulose, and lignin, accounting for 60–90% of entire dry weight; other components include extractives, protein, and pectin. Thermochemical or biological techniques [1, 3] have been carried out aiming at the conversion of biomass for strategic utilization. Biomass-based fuels, such as bioethanol, biogas, biodiesel, and biooil, have been used as complementary fuels for decades; meanwhile, various value-added chemicals, such as furans, aldehydes, ketones, and aromatics, so-called platform compounds, can also be produced in biorefinery plants [1–3].

Extractives are natural nonstructured nonpolymer components in biomass and are capable of being extracted with water and organic solvents [4]. Chemically, extractives cover a wide range of species, such as aliphatic hydrocarbons, alcohols, acids, fats, terpenes, steroids, resin acids, rosin, phenols, waxes, glycosides, quinines, and proteins, accounting for 5–30% of dry weight of biomass [4]. The components of the extractives from biomass vary significantly [5] according to the family, genera, and species and even the location, part, age, and season.

Although the extractives are considered to be nonstructural substances with low contents, they play an important role in the attractive forces of adjacent biomass particles [6] by providing weak H-bonding and van der Waal's forces [7, 8]. Furthermore, extractives may affect subsequent characterization, further degradation processes, and even the utilization of carbohydrates and lignin in biomass. For example, due to incomplete removal in the extraction, residual extractives will precipitate together with insoluble lignin, leading to an overestimation of lignin content [9]. In pulping process, serious pitch problem may arise if extractives are not removed effectively; for example, sterols and waxes are insoluble and will deposit in alkaline solutions [5]. In thermochemical conversion of biomass, extractives are decomposed under lower temperature avoiding significant influence on product properties. However, extractives can catalyze the reactions involved in the pyrolysis of biomass [10] and additional products derived from extractives can alter the distribution of the final products [11]. Furthermore, some extractives are potentially toxic to microorganisms which would negatively influence the biochemical conversion of biomass [12]. Therefore, it is necessary to remove them prior to downstream analysis or handling of biomass [13].

The removal of extractives depends heavily on the solvents and conditions used. Generally, water and ethanol extractives contain organic acids, inorganic substances, waxes, nonstructural sugars, and so forth. [14, 15]. Hot water can extract tannins and phenolics effectively from biomass [16–18], while ethanol, acetone, and dichloromethane can remove phytosterols and lipophilic extractives from wood significantly, but leaving fatty acid esters with considerable amounts [11]. Petroleum ether (PE) and carbon disulfide (CS_2_) are organic solvents with low polarities. They can extract alkanes, waxes, benzene-ring containing compounds (BRCCs), fatty acids, and organonitrogen compounds (ONCs) effectively from degraded biomass samples [19–22]. Typical components in extractives can also be enriched in the extraction fractions. PE is effective for the removal of alkanes and waxes in stalks [19] and for the extraction of BRCCs in biooil produced from pyrolysis of rice husk [20]. CS_2_ was reported to have strong *π*-*π* interaction with fatty acids [21] and ONCs in the extraction [22].

A systematic study showed that wheat straw (WS) was more readily depolymerized with the oxidation of NaOCl aqueous solution after sequential extraction with several organic solvents [23]. Oxidative degradation of organic components in WS might be hindered by extractives to some extent. In order to explore more information on the extraction process, in this investigation, PE and CS_2_ were used as the solvents in the sequential extraction of WS, and detailed characterization of the extraction fractions was carried out. Fourier transform infrared (FTIR) spectroscopy, as a routine technique for analysis of organic substances, was used to determine functional groups in extraction fractions. Difference between WS and extracted WS in their components could be clearly presented. The volatile species in extractives were identified and characterized with gas chromatography/mass spectrometry (GC/MS). GC/MS is a frequently used technique to identify compounds based on the high effective chromatographic separation of species with different volatilities. Mass spectrometer provides high resolution for charged fragments derived from molecules. FTIR and GC/MS are convenient and reliable methods for the analysis of complex samples, such as the degradation products from biomass and coals [24, 25]. The chemical states of elements contained in extractives were assayed by X-ray photoelectron spectroscopy (XPS). Binding types between atoms can be assigned by calculated binding energy. Though it is very expensive to conduct, XPS analysis is useful in understanding the composition of samples by providing fundamental structural information [26, 27]. Transmission electron microscopy (TEM) coupled with energy dispersive spectrometry (EDS) and electron probe microanalysis (EPMA) were employed for the determination of abundance of organic/inorganic atoms in the extraction fractions. TEM-EDS method provides accurate qualitative and quantitative analyses for elements with atomic number from 4 (Be) to 92 (U). The costs of TEM-EDS and EPMA analyses are higher than FTIR and GC/MS analyses since they contain relatively more expensive instruments. They were more often used in elemental analysis of inorganic materials, such as semiconductors and cells. In recent years, EDS was used in the characterization of lignite and its alkali extracted residue [28]. Very few reports were issued on the analysis of inorganic matters derived from biomass [29, 30].

## 2. Experimental

### 2.1. Materials

WS was purchased from Xuzhou, Jiangsu Province, China. It was washed with distilled water for several times to remove sandy soil and then dried in sunlight for more than two months. Then the dried WS was chopped into small pieces and pulverized to pass through an 80-mesh sieve (<180 *μ*m) followed by desiccation in a vacuum drying oven at 80°C for 48 h.  Table 1 shows the proximate and ultimate analyses of the dried WS sample. PE and CS_2_ were of analytical purity and distilled at their boiling points under atmospheric pressure with a Büchi R-134 rotary evaporator to avoid contaminative impurities.

### 2.2. Extraction

50.0 g WS sample and 500 mL PE were added to a 1 L beaker, magnetically stirred at 25°C for 1 h, and then placed in a thermostatic ultrasonic bath set at 25°C for about 6 h. The mixture was filtrated to afford a filter cake EFC_1_ and a filtrate EF_PE1_. Then the EFC_1_ was extracted by another 500 mL of PE with the same procedure previously used, affording a filter cake EFC_2_ and a filtrate EF_PE2_. The two filtrates were combined together to afford a solution EF_PE_. EFC_2_ was dried in a vacuum drying oven at 40°C for 48 h to remove the solvent to a constant weight (±0.01 g). The amount of extractives extracted by PE was calculated as the weight loss based on the initial dried WS.

Then EFC_2_ was extracted by CS_2_ with the same procedure used for PE to afford a solution EF_CS2_ and a filter cake EFC_4_. The same drying conditions were used for EFC_4_. The amount of extractives extracted by CS_2_ was calculated as the weight loss based on the difference between EFC_2_ and EFC_4_. EF_PE_ and EF_CS2_ were concentrated to remove the solvents drastically by using a Büchi R-134 rotary evaporator while keeping the solvent temperature at 40°C under vacuum distillation condition. The dried extractives from EF_PE_ and EF_CS2_ were stored in a desiccator at 25°C.

### 2.3. FTIR Analysis

The WS sample, extraction residue EFC_4_, and the extractives from EF_PE_ and EF_CS2_ were analyzed with a Nicolet Magna IR-560 FTIR spectrometer using KBr pellet method. The spectra were recorded by collecting 50 scans at a resolution of 8 cm^−1^ in reflectance mode with a measuring region of 4000–500 cm^−1^.

### 2.4. GC/MS Analysis

The extractives were analyzed with a Hewlett-Packard 6890/5973 GC/MS system equipped with a HP-5MS capillary column (cross link 5% PH ME siloxane, 60 m × 0.25 mm* i.d.*, 0.25 *μ*m film thickness) and a quadrupole analyzer. The compounds were ionized by electron ionization under 70 eV. Quadrupole mass analyzer was used to obtain the mass spectra. Helium was used as the carrier gas with a flow rate at 1.0 mL/min. The column was heated first at a rate of 5°C/min from 60°C to 150°C and then at a rate of 7°C/min from 150°C to 300°C (and held at 300°C for 40 min). Both injector and detector temperatures were set at 300°C. The scanned mass range was 30–500* m/z*. The reproducibility of quantitative analysis for the species was conducted by duplicated injection of the samples. The data were acquired and processed using Chemstation software together with GC/MS system. The species were identified by comparing mass spectra with National Institute of Standards and Technology (NIST) library data according to fragmentation rules of organic species under electron ionization condition.

### 2.5. XPS Analysis

The XPS analysis was performed on an ESCALAB 250Xi system (Thermo-Fisher, USA). The source gun type was Al K Alpha, and spot size was 900 *μ*m. Data were recorded by collecting 30 scans in 20 min with pass energy at 20.0 eV. Survey scan was conducted within the range 0–1000 eV. Peak fitting was used for the spectra to the assignment of different chemical bonds according to specific binding energy.

### 2.6. TEM-EDS Analysis

EF_PE_ sample was dispersed with ethanol, then sprayed with carbon film and loaded on 300-mesh copper grids under sonic condition for 10 min, and then dried under lamp. TEM analysis was performed on a Tecnai G^2^ F20 electron microscope (FEI, USA) at an accelerating voltage of 200 kV and linked to an X-ray analysis system (Oxford EDS 6767). Determination of C atoms was interfered by carbon film introduced in the preparation of the sample. O element was not taken into consideration in the TEM-EDS analysis since previous methods provided detailed results. Inorganic elements with trace amounts in extractives were assayed, such as Al, Si, P, S, Cl, and Ca atoms.

### 2.7. EPMA Analysis

In order to find more information on the elemental composition of EF_PE_ and to verify the results obtained with TEM-EDS analysis, EPMA analysis was carried out, especially concentrating on K, Na, Mg, S, and Ca elements. The analysis was conducted on an 8050G system (Shimadzu, Japan). Schottky emission mode was used, and the accelerating voltage was 15 kV.

## 3. Results and Discussions

### 3.1. Extraction Yields

The dried EFC_4_ lost 2.48 g comparing to the WS sample, indicating that the extractives accounted for 4.96% of the starting material. PE and CS_2_ were removed completely with a rotary evaporator under reduced pressure. The weights of extractives from EF_PE_ and EF_CS2_ were 1.66 g and 0.82 g, respectively. Waxes and BRCCs could be concentrated into EF_PE_ because PE was effective for dewaxing of stalks [19] and for the extracting of BRCCs [20]. Species containing double bond or triple-bond, such as fatty acids, aldehydes, ketones, and ONCs, could be enriched into EF_CS2_ due to their strong *π*-*π* interaction with CS_2_ [21, 22].

### 3.2. FTIR Analysis

**Figure 1:**
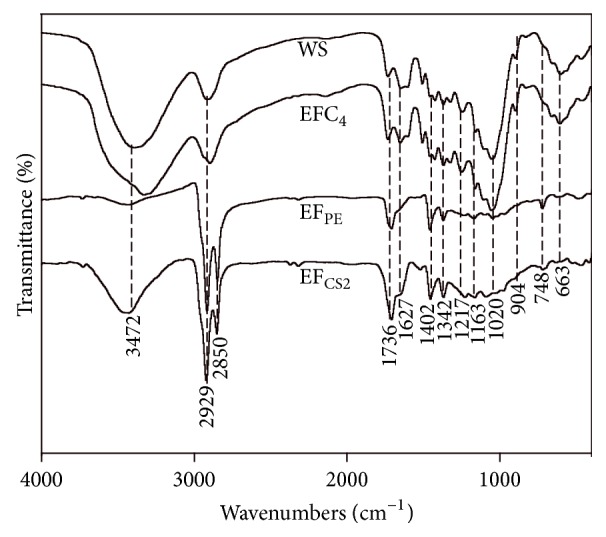
FTIR analysis of WS, EFC_4_, EF_PE_, and EF_CS2_.

Significant differences were observed in the FTIR spectra of WS sample, EF_PE_, EF_CS2_, and EFC_4_, as shown in Figure 1. Wide and strong peaks around 3200–3700 cm^−1^ were attributed to the vibration of -OH; for example, peaks 3450–3650 cm^−1^ and 3200–3400 cm^−1^ were assigned to free and associated -OH, respectively. Except for EFC_4_, all the other three samples showed wide peak around 3500 cm^−1^, implying the presence of species containing free -OH. There was an interesting transformation of absorbance peak before and after the extraction, that is, from free -OH to associated -OH. Species containing free -OH in extractives can be extracted from WS sample with PE and CS_2_, sequentially. Free -OH containing species were enriched in EF_CS2_ rather than EF_PE_. Peak around 3300–3500 cm^−1^ in the spectrum of EF_CS2_ could also be attributed to the vibration of -NH, implying the presence of ONCs [22]. However, in spectrum of EF_PE_, only a weak peak was obtained within this range. Peaks around 2929 cm^−1^ and 2850 cm^−1^ were attributed to the stretching of -CH_2_- and -CH_3_, implying the presence of alkanes, waxes, aliphatics, and so forth. Fine structures of spectra were only observed for the two extracts, namely, EF_PE_ and EF_CS2_, implying the selectivity of these two solvents for -CH_2_- and -CH_3_ containing species. The intense peaks around 1736 cm^−1^ and 1627 cm^−1^ were attributed to the vibration of C=O, indicating the presence of aldehydes, ketones, carboxylic acids, esters, and so forth [31]. All the four samples contained the peak at 1736 cm^−1^ with similar intensity. However, the peaks at 1627 cm^−1^ were weaker in spectra of EF_PE_ and EF_CS2_. Peak around 1402 cm^−1^ could be assigned to the bending of C-H in aliphatics, O-H in carboxylic acids, C-O-H in alcohols, and so forth. This peak was common in each spectrum of samples. Peaks around 1736 cm^−1^, 1627 cm^−1^, and 1402 cm^−1^ could also be assigned to characteristic absorption of ONCs, namely, amide I and II bands and -NO_2_ stretching, respectively [22]. Peaks at 1163 cm^−1^, 1020 cm^−1^, and 663 cm^−1^ were assigned to stretching of C-O-C and in-plane bending of aromatic C-H, which were observed obviously in the spectra of WS and EFC_4_. The C-O-C structure can be found in almost all kinds of hemicelluloses and cellulose in biomass and the aromatic C-H present in lignins. In the spectra of EF_PE_ and EF_CS2_, peaks around 748 cm^−1^ were attributed to the stretching of -CH_2_- in waxes and aliphatics [32]. The difference between WS and EFC_4_ spectra was difficult to figure out; meanwhile EF_PE_ and EF_CS2_ shared very similar spectrum. Significant differences between the two groups (WS/EFC_4_ and EF_PE_/EF_CS2_) lie in peaks around 3200–3700 cm^−1^ and 1020 cm^−1^, indicating different states of -OH and presence of aromatic C-H.

### 3.3. GC/MS Analysis of the Species in the Extracts

**Figure 2:**
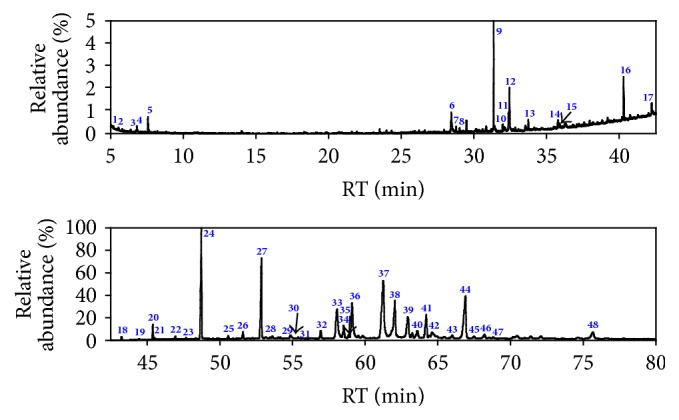
TICs of EF_PE_ from WS.

**Figure 3:**
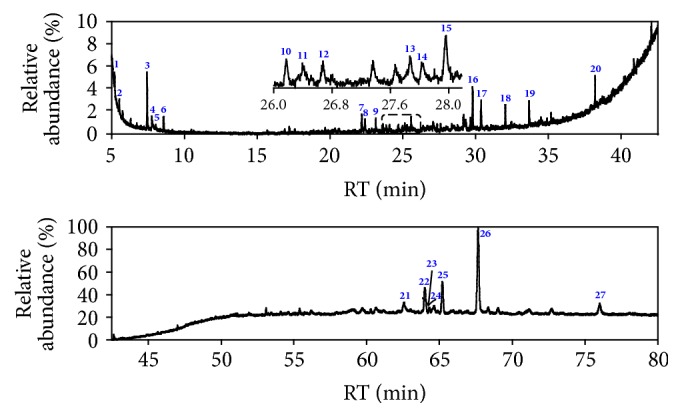
TICs of EF_CS2_ from WS.

**Figure 4:**
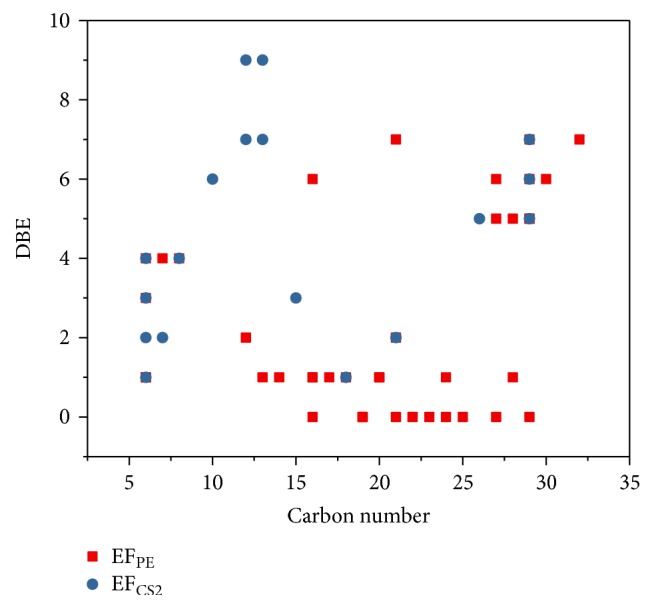
The relationship between DBE and carbon number for compounds identified in EF_PE_ and EF_CS2_.

In total, 48 and 27 compounds were identified in EF_PE_ and EF_CS2_, respectively, with GC/MS analysis. The total ionic chromatograms (TICs) were presented in Figures 2 and 3, respectively. These species can be classified into alkanes, alkenes, arenes, alcohols, furan, aldehydes, ketones, ONCs, carboxylic acid (CA), esters, and others, as listed in Tables S1 and S2 (see Supplementary Material available online at https://doi.org/10.1155/2017/7305682). Double bond equivalent (DBE) was calculated for each species identified in EF_PE_ and EF_CS2_; and the plot of DBE versus carbon number was used to demonstrate the degrees of unsaturation of compounds, shown in Figure 4. From the plot, one can observe that compounds were roughly divided into two groups in these two samples, namely, lower DBE group and higher DBE group. CS_2_ favors species with higher DBEs and less carbon numbers, while species with more carbon numbers and lower DBEs were enriched in PE.

**Figure 5:**
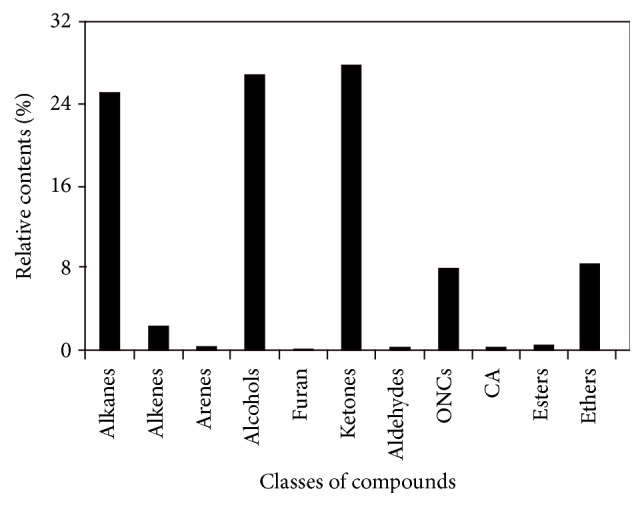
Relative contents of classes of compounds identified in EF_PE_.

Various long-chain alkanes, alkenes, and aldehydes derived from waxes were identified, which could be extracted easily by PE [19]. Two arenes were identified in EF_PE_ (**1** and** 3**). Eight alkanes with carbon numbers ranging from 16 to 29 were detected. All the 5 alcohols identified were sterols. Only one furan was identified, that is, 2,5-dimethylfuran** (2)**, which could be derived from cellulose and hemicelluloses [33]. For the contents of the classes of compounds identified in EF_PE_, ketones, alkanes, alcohols, and ONCs were abundant, as shown in Figure 5. Ketones were the most abundant species in EF_PE_. Besides alkanones (**11** and** 12**) and furanone** (16)**, abundance sterones were identified (**35**,** 39**–**42**,** 44**–**46,** and** 48**). Sterols and sterones are typical species in extractives with considerable amounts, which can be extracted easily with solvents. Extractives were considered as potential material for production of medicine for pharmaceutical utilization based on the steroids components, such as sterols and sterones [34]. Sterones tablets containing testosterone, norethindrone, methandienone, and so forth have been used for treatment of many diseases. There were 17 steroids identified in the EF_PE_, accounting for about 64.52% in relative content, such as cholests and stigmasts. Among them, the most abundant were 22,23-dihydrostigmasterol** (37)** and stigmastane-3,6-dione** (44)**.* N*,4-Dimethylbenzenesulfonamide** (6)** was the only one sulfur-containing species identified. Two of the esters identified, namely, butyl methyl phthalate** (10)** and dipropyl phthalate** (13)**, might be impurities coming from the GC/MS system with plastic or rubber devices or contaminants introduced in the process of sample preparation [35].

**Figure 6:**
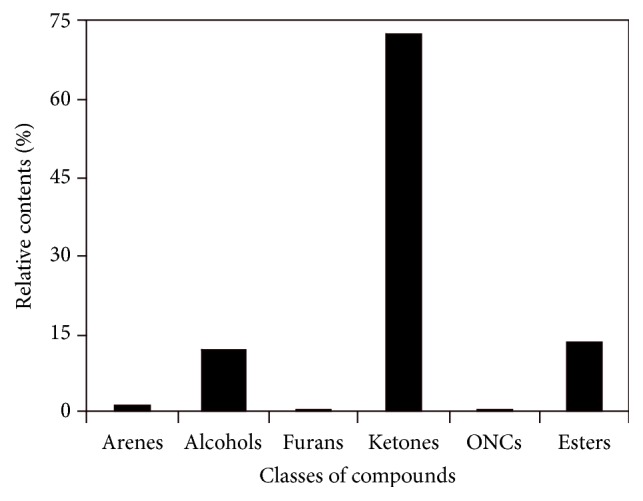
Relative contents of classes of compounds identified in EF_CS2_.

Compounds identified in EF_CS2_ with GC/MS analysis can be classified into arenes, alcohols, furans, ONC, ketones, and esters, as listed in Table S2. Neither alkanes nor alkenes were identified in the EF_CS2_, and no species that could be derived from waxes were found. Besides benzene** (1)** and xylene** (5)**, acenaphthene** (9)** and 9H-fluorene** (14)** were identified and grouped into the arenes. All the three alcohols identified were steroids (**15**,** 21,** and** 23**). 2,5-Dimethylfuran** (2)** and dibenzofuran** (11)** may be derived from the hemicellulose and lignin, respectively. Similar with EF_PE_, ketones were the most abundant class identified in EF_CS2_ and could be classified further into alkanones (**3** and** 16**), cycloalkanone** (6)**, furanone** (20)**, alkenone** (8),** and sterones** (24–27)**. Seven steroids were identified in the EF_CS2_ with relative content of 79.58%. The four phthalates identified (**7**,** 13**,** 17,** and** 18**) might not be the components in the extractives, which could be considered as impurities or contaminants [35]. The relative contents of the classes of compounds identified in EF_CS2_ were shown in Figure 6. Ketones, esters, and alcohols were the three most abundant classes.

### 3.4. XPS Analysis

**Figure 7:**
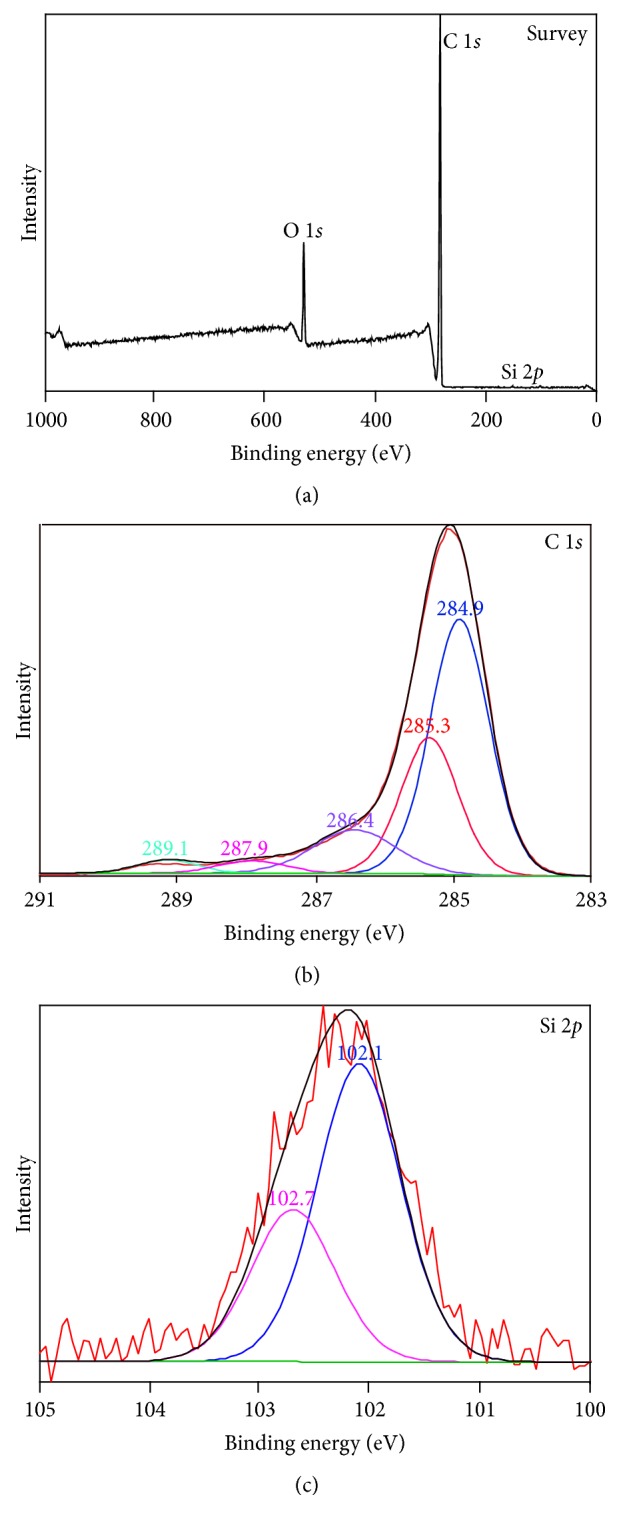
XPS analysis of EF_PE_. ((a) survey scan of the sample; (b) peak fitting of the C 1*s*; and (c) peak fitting of the Si 2*p*.)

As displayed in Figure 7, 101.8 eV, 284.1 eV, and 531.9 eV were attributed to Si 2*p*, C 1*s*, and O 1*s* [36], respectively, implying the presence of the Si-, C-, and O-containing species in EF_PE_. Peak fittings for C 1*s* and Si 2*p* were conducted (see (b) and (c) in Figure 7) and the resulting peaks were assigned for different chemical bonds indicating the status of C, O, and Si atoms according to their special binding energy (BE), as shown in Table 2. The full width at half maximum (FWHM) of each chemical bond was also calculated. According to the atomic percentage for specific chemical bond, C atoms contained in C-C bond accounted for 48.54% indicating the presence of high amount of long-chain aliphatic species, such as alkanes, waxes, and/or steroids, which is consistent with the componential analysis by GC/MS. C-COOR containing species could be attributed to fatty acids and esters holding 25.29% of C atoms. Other types of C atoms were in C-O, C=O, and O-C=O structures [36]. Si-O bond was divided into two bonds in peak fitting, namely, 102.1 eV and 102.7 eV, which could be assigned to Si_2_O_3_ and SiO_2_, respectively. XPS is a surface-sensitive technique by providing accurate qualitative and quantitative analyses of elemental composition, chemical state, and electronic state of the elements in a sample. It can be used as complementary method along with FTIR and GC/MS analyses to characterize complex samples derived from biomass.

### 3.5. TEM-EDS Analysis

As shown in Figure S1 (Supporting Information), two areas (Areas 1 and 2) were selected for the scanning and determining elements with TEM-EDS in EF_PE_. Data were collected from six points in each area. Carbon film was used in the sample preparation, and it would interfere with the identification of C atoms. C and O elements were not considered in the detection with TEM-EDS analysis. Al, Si, P, S, Cl, and Ca atoms were counted on the data point of EF_PE_ and the results were listed in Table 3. Ca, Si, and S atoms were counted with higher intensities for both areas, which may present in inorganic materials or complexes. Si is known as beneficial element for wheat straw [37, 38]. Morphological silica materials, such as SiO_2_, can be obtained by thermochemical treatments [39]. Phytoliths are the main forms of Si uptaken by wheat straw [40]. Then the Si-containing species deposit within different intracellular and extracellular structures. Ca atoms were detected in the extractives with comparable accounts to Si. Calcium oxalate is another main component in phytoliths [41, 42] together with Si-containing species. The concentration of Si in WS ranges from 1.5 to 12 g/kg [43], while Ca content in WS is around 5.6 g/kg [44]. Current application of the Ca and Si in biomass was concentrated on the production of ash and further for the preparation of value-added materials, such as catalyst [45]. By using organic solvents in the extraction, namely, PE and CS_2_, only trace amounts of Si and Ca were extracted since most of them were in their inorganic forms. S atoms were also counted with similar amounts to Si and Ca atoms near Area 2. The content of S in WS was in the range of 0.1–0.7% (air dried basis) [46, 47]. Sulfur atom is building block of some proteins and a key ingredient in the formation of chlorophyll. Most of the sulfur is assimilated by the roots in the form of SO_4_^2−^. Then it is stored in the form of sulfate and metabolized and/or incorporated into organic structures. It was reported that almost all the sulfur in rice hull occurs in organic form [48]. However, only one sulfur-containing compound was detected in GC/MS analysis of extracts (*N*,4-dimethylbenzenesulfonamide in EF_PE_, see Table S1 in Supporting Information). In biomass, most of Al, P, and Cl atoms are in their inorganic forms. Among them, the existence of P atoms could be attributed to phosphate in biomass. Phosphate fertilizer is important for the growth of plants and will be stored mainly in the form of phosphate.

In order to obtain more accurate information of inorganic substances, more data points should be collected; however, due to the intrinsic limitation of TEM-EDS analysis, only qualitative and semiquantitative results can be obtained. The detection of inorganic substances with trace amounts relies on the progress of in situ analytical techniques with higher resolutions. Meanwhile, enlarging the number of data points is necessary to achieve more accurate and representative analytical results.

### 3.6. EPMA Analysis

Similar qualitative results for trace amount of elements were obtained by EPMA. Eight data points were selected for EF_PE_. Sample image and net intensities for C, Na, Mg, Si, S, K, and Ca atoms of each data point were presented in Figure S2 (Supporting Information). In EPMA, metal elements K, Na, and Mg were identified well in the EF_PE_. Metal ions are usually in the forms of oxides presented in ash after the combustion of biomass [49]. K and Na atoms, in their forms of cations, are crucially important nutrients affecting most of the biochemical and physiological processes to promote plant growth and metabolism [50, 51]. Mg is one of the most important nutrients to plants. It provides the central ions of chlorophyll to accomplish photosynthesis in plants and is involved in many enzyme activities and the structural stabilization of tissues [52]. Due to intrinsic restriction, similar with TEM-EDS analysis, EPMA provides qualitative and semiquantitative results of inorganic elements.

## 4. Conclusions

The PE and CS_2_ extractable extractives were weighted up to 4.96% of initial WS material. Detailed componential characterization of the extractives was carried out by FTIR, GC/MS, TEM-EDS, EPMA, and XPS. FTIR and GC/MS analyses can be treated as universal techniques used in the compositional characterization of complex samples containing various organic species. PE and CS_2_ were proved to be effective solvents for extraction of waxes, ketones, esters, and ONCs. Among the detected compounds with GC/MS, ketones were the most abundant species in the two extraction fractions. Other classes of species were alkanes, alkenes, alcohols, aldehydes, ONCs, and so forth. Considerable amounts of steroids especially sterones were identified. This kind of species could be used for medicine production. Trace amounts of atoms including Ca, Si, K, Cl, Na, and S were counted with EDS in extractives. EPMA provided similar results with TEM-EDS analysis. Chemical bonds and their abundances were assigned for C, O, and Si by using XPS analysis. Most of the C atoms in the species of extractives were contained in the structures of C-C, C-COOR, and C-O. Si atoms could be assigned in Si-O structures contained in Si_2_O_3_ and SiO_2_.

FTIR analysis can be used to determine the functional group changes in the sample before and after extraction, and GC/MS analysis is suitable for the detection of volatile organic species providing more accurate information of individuals. These two techniques were used conventionally and effectively in the compositional analysis of complex samples. TEM-EDS and EPMA analyses are nondestructive techniques providing detailed information on the composition of elements and the contents of various atoms, especially for inorganic elements rather than C atoms. By calculating the BEs of atoms in specific chemical bonds, XPS analysis can provide fundamental structural information that is useful for understanding the composition of complex samples. Generally, the later three analytical methods are more expensive than the former two. Based on the intrinsic properties of the samples in this investigation, FTIR, GC/MS, and XPS were recommended methods used in industries. Comprehensive understanding of the composition of extractives and other complex samples relies on the combination of the advanced analytical techniques.

## Supplementary Material

Table S1: Compounds identified in EF_PE_ with GC/MS.
Table S2: Compounds identified in EF_CS2_ with GC/MS.
Figure S1: TEM-EDS analysis of EF_PE_ (images of sample Areas 1 and 2; and results for data points 1-6 for each Area, respectively).
Figure S2: EPMA analysis of EF_PE_ (image of sample area; and results for data points 1-8, respectively).

## Figures and Tables

**Table 1:** Proximate and ultimate analyses (wt.%) of WS.

Proximate analysis			Ultimate analysis				
*M*_ad_	*A*_d_	*V*_daf_	C	H	O^*∗*^	N	S

8.0	8.2	70.2	42.3	6.6	50.2	0.3	0.6

^*∗*^By difference: *M*_ad_, moisture on air dried basis; *A*_d_, ash on dry basis; *V*_daf_, volatile matter on dry and ash-free basis.

**Table 2:** Chemical statuses of C and Si atoms in EF_PE_ analyzed with XPS.

Chemical bond	BE (eV)	FWHM (eV)	Area	Atomic (%)
C-C	284.9	1.01	34722.3	48.54
C-COOR	285.3	0.98	18089.1	25.29
C-O	286.4	1.42	8333.9	11.66
-C=O	287.9	1.13	1899.8	2.66
O-C=O	289.1	1.01	1779.9	2.49
Si-O	102.1	0.91	166.8	0.38
	102.7		85.1	0.01

**Table 3:** TEM-EDS analysis of elements and their percentages of weight (wt.%) in EF_PE_.

Areas	Data points	Al (K)	Si (K)	P (K)	S (K)	Cl (K)	Ca (K)
1	1	32.64	Nd	28.54	30.67	8.12	Nd
	2	3.11	4.78	Nd	Nd	Nd	92.09
	3	3.60	16.91	Nd	0.36	Nd	79.11
	4	10.72	15.50	Nd	Nd	Nd	73.77
	5	Nd	75.96	10.70	Nd	13.32	Nd
	6	Nd	61.01	6.53	11.06	21.39	Nd
	Average	8.35	29.03	7.63	7.02	7.14	40.83

2	1	3.65	16.96	Nd	38.71	5.43	35.22
	2	Nd	4.85	0.33	46.20	Nd	48.60
	3	Nd	47.59	49.64	Nd	Nd	2.76
	4	Nd	49.74	9.59	19.68	9.20	11.76
	5	13.09	Nd	Nd	37.81	Nd	49.08
	6	Nd	61.88	21.15	16.96	Nd	Nd
	Average	2.79	30.17	13.45	26.56	2.44	24.57

Nd: not detected.
