# Supplementary material for: Detailed Componential Characterization of Extractable Species with Organic Solvents from Wheat Straw
Source: Int J Anal Chem. 2017 Nov 1;2017:7305682. doi: 10.1155/2017/7305682 (PMC5676445; doi:10.1155/2017/7305682)
Supplement: Supplementary file 1 — Table S1: Compounds identified in EFPE with GC/MS. Table S2: Compounds identified in EFCS2 with GC/MS. Figure S1: TEM-EDS analysis of EFPE (images of sample Areas 1 and 2; and results for data points 1-6 for each Area, respectively). Figure S2: EPMA analysis of EFPE (image of sample area; and results for data points 1-8, respectively). [file 7305682.f1.docx]

Table S1. Compounds identified in EF_PE_ with GC/MS.

| No. | Compounds | Formula | Relative contents (%) |
| --- | --- | --- | --- |
| **Alkanes** |  |  |  |
| **15** | Hexadecane | C_16_H_34_ | 0.019 |
| **17** | Nonadecane | C_19_H_40_ | 0.056 |
| **22** | Heneicosane | C_21_H_44_ | 0.256 |
| **24** | Docosane | C_22_H_46_ | 11.82 |
| **25** | Tricosane | C_23_H_48_ | 0.322 |
| **27** | Pentacosane | C_25_H_52_ | 9.982 |
| **31** | Heptacosane | C_27_H_56_ | 0.24 |
| **34** | Nonacosane | C_29_H_60_ | 2.132 |
| **Alkenes** |  |  |  |
| **8** | 2,10-Dimethyl-6-methyleneundecane | C_14_H_28_ | 0.018 |
| **14** | 1-Hexadecene | C_16_H_32_ | 0.064 |
| **21** | 1,19-Docosadiene | C_20_H_38_ | 0.285 |
| **26** | 1-Tetracosene | C_24_H_48_ | 0.695 |
| **32** | 7-Octacosene | C_28_H_56_ | 1.132 |
| **Arenes** |  |  |  |
| **1** | Benzene | C_6_H_6_ | 0.014 |
| **3** | Toluene | C_7_H_8_ | 0.012 |
| **Alcohols** |  |  |  |
| **20** | 1-Nonadecanol | C_19_H_40_O | 0.901 |
| **30** | Cholest-5-en-3-ol | C_27_H_46_O | 0.718 |
| **33** | Brassicasterol | C_28_H_48_O | 7.506 |
| **37** | 22,23-Dihydrostigmasterol | C_29_H_50_O | 16.705 |
| **43** | Taraxasterol | C_30_H_50_O | 1.554 |
| **Furan** |  |  |  |
| **2** | 2,5-Dimethylfuran | C_6_H_8_O | 0.01 |
| **Aldehydes** |  |  |  |
| **4** | Hexanal | C_6_H_12_O | 0.026 |
| **7** | Tridecanal | C_13_H_26_O | 0.021 |
| **19** | Octadecanal | C_18_H_36_O | 0.113 |
| **23** | Phytol | C_20_H_40_O | 0.168 |
| **Ketones** |  |  |  |
| 5 | 4-Hydroxy-4-methyl-2-pentanone | C_6_H_12_O_2_ | 0.085 |
| 9 | 7-Hexyloxepan-2-one | C_12_H_22_O_2_ | 0.37 |
| 11 | 3-Hexadecanone | C_16_H_32_O | 0.062 |
| 12 | 2-Heptadecanone | C_17_H_34_O | 0.158 |
| 16 | 5-Methyl-5-(4,8,12-trimethyltridecyl)dihydro-2(3H)-furanone | C_21_H_40_O_2_ | 0.157 |
| 35 | Pregn-4-ene-3,20-dione | C_21_H_30_O_2_ | 0.625 |
| 39 | Cholest-4-en-3-one | C_27_H_44_O | 5.089 |
| 40 | Rhapontisterone | C_27_H_44_O | 1.827 |
| 41 | 4,22-Stigmastadiene-3-one | C_29_H_46_O | 4.284 |
| 42 | Spinasterone | C_29_H_46_O | 0.303 |
| 44 | Stigmasta-4,24(28)-dien-3-one | C_29_H_46_O | 11.265 |
| 45 | Stigmasta-3,5-dien-7-one | C_29_H_46_O | 0.495 |
| 46 | Stigmast-4-en-3-one | C_29_H_48_O | 0.962 |
| 48 | Stigmastane-3,6-dione | C_29_H_48_O_2_ | 2.441 |
| **ONCs** |  |  |  |
| 6 | *N*,4-Dimethylbenzenesulfonamide | C_8_H_11_NO_2_S | 0.127 |
| 38 | *N*-[2-(4-Morpholinyl)ethyl)-α-hydroxybenzenebutanamide | C_16_H_24_N_2_O_3_ | 7.892 |
| **CA** |  |  |  |
| 18 | 2-((2-Ethylhexyloxy)carbonyl)benzoic acid |  | 0.241 |
| **Esters** |  |  |  |
| 10 | Butyl methyl phthalate | C_13_H_16_O_4_ | 0.028 |
| 13 | Dipropyl phthalate | C_14_H_18_O_4_ | 0.049 |
| 47 | Lup-20(29)-en-3-yl acetate | C_32_H_52_O_2_ | 0.36 |
|  |  |  |  |
| **Others** |  |  |  |
| 28 | 1-(Hexyloxy)octadecane | C_24_H_50_O | 0.337 |
| 29 | 4,4-Dimethyl-cholesta-6,22,24-triene | C_29_H_46_ | 0.126 |
| 36 | Stigmastan-3,5-diene | C_29_H_48_ | 7.746 |

Table S2. Compounds identified in EF_CS2_ with GC/MS.

| No. | Compounds | Formula | Relative contents (%) |
| --- | --- | --- | --- |
| **Arenes** |  |  |  |
| **1** | Benzene | C_6_H_6_ | 0.36 |
| **5** | Xylene | C_8_H_10_ | 0.185 |
| **9** | Acenaphthene | C_12_H_10_ | 0.307 |
| **14** | 9H-Fluorene | C_13_H_10_ | 0.225 |
| **Alcohols** |  |  |  |
| **15** | Rosifoliol | C_15_H_26_O | 0.411 |
| **21** | Sitosterol | C_29_H_50_O | 9.567 |
| **23** | 4,4-Dimethyl-cholest-5-en-3-ol | C_29_H_50_O | 1.991 |
| **Furans** |  |  |  |
| **2** | 2,5-Dimethylfuran | C_6_H_8_O | 0.224 |
| **11** | Dibenzofuran | C_12_H_8_O | 0.295 |
| **Esters** |  |  |  |
| **7** | Dimethyl phthalate | C_10_H_10_O_4_ | 0.438 |
| **13** | Diethyl phthalate | C_12_H_14_O_4_ | 0.292 |
| **17** | Butyl ethyl phthalate | C_14_H_18_O_4_ | 0.66 |
| **18** | Dibutyl phthalate | C_16_H_22_O_4_ | 0.513 |
| **19** | Isopropyl 2-phenyl-4,5-dihydrooxazole-4-carboxylate | C_13_H_15_NO_3_ | 0.756 |
| **22** | 3,7,11,15-Tetramethylhexadecyl nicotinate | C_26_H_45_NO_2_ | 11.556 |
| **Ketones** |  |  |  |
| **3** | 2-Hexanone | C_6_H_12_O | 1.032 |
| **4** | 4-Hydroxy-4-methyl-2-pentanone | C_6_H_12_O_2_ | 0.816 |
| **6** | Cyclohexanone | C_6_H_10_O | 0.342 |
| **8** | 5-Methylhex-4-en-3-one | C_7_H_12_O | 0.261 |
| **10** | 4-(4-Chlorophenyl)but-3-en-2-one | C_10_H_9_ClO | 0.202 |
| **16** | 6,10,14-Trimethylpentadecan-2-one | C_18_H_36_O | 0.868 |
| **20** | 5-Methyl-5-(4,8,12-trimethyltridecyl)dihydrofuran-2(3H)-one | C_21_H_40_O_2_ | 0.803 |
| **24** | 24-Ethyl-5α-cholesta-2,22-dien-6-one | C_29_H_46_O | 4.373 |
| **25** | Stigmast-4-en-3,6-dione | C_29_H_46_O_2_ | 13.14 |
| **26** | Stigmasta-4,24(28)-dien-3-one | C_29_H_46_O | 39.148 |
| **27** | 17-(1,5-Dimethylhexyl)-2-(1-hydroxyethylidene)-10,13-  dimethylhexadecahydrocyclopenta[a]phenanthren-3-one | C_29_H_48_O_2_ | 11.032 |
| **ONC** |  |  |  |
| **12** | 1-Methylpyrrolidin-2-one | C_5_H_9_NO | 0.201 |


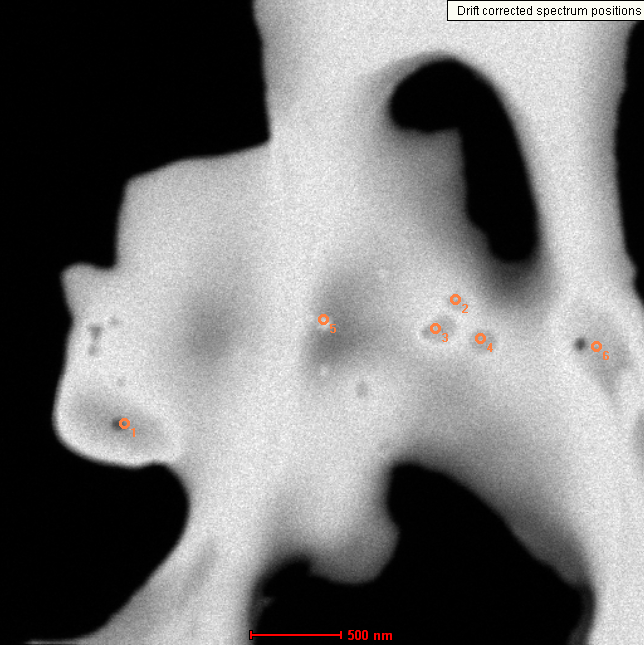


(Continued)

(Continued)

Figure S1. TEM-EDS analysis of EF_PE_. Area 1: TEM image shows the location of data points; following 6 EDS plots present the intensities of different atoms of each point.


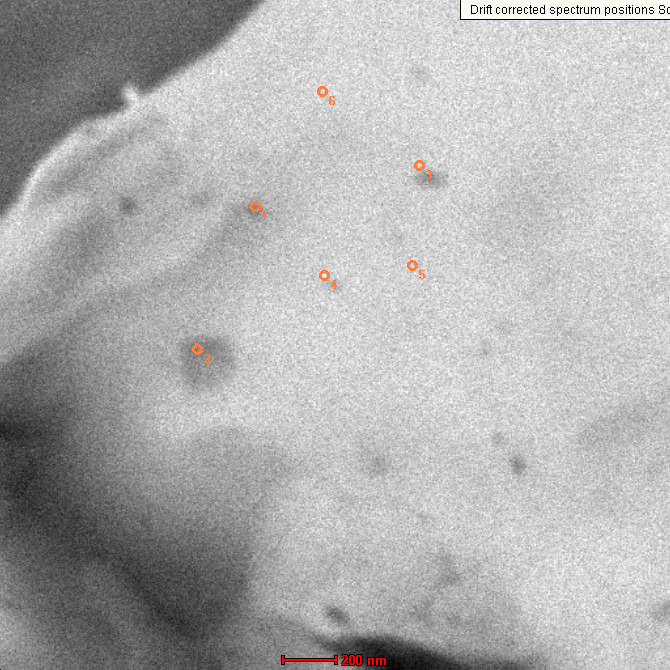


(Continued)

Figure S1. TEM-EDS analysis of EF_PE_. Area 2: TEM image shows the location of data points; following 6 EDS plots present the intensities of different atoms of each point.


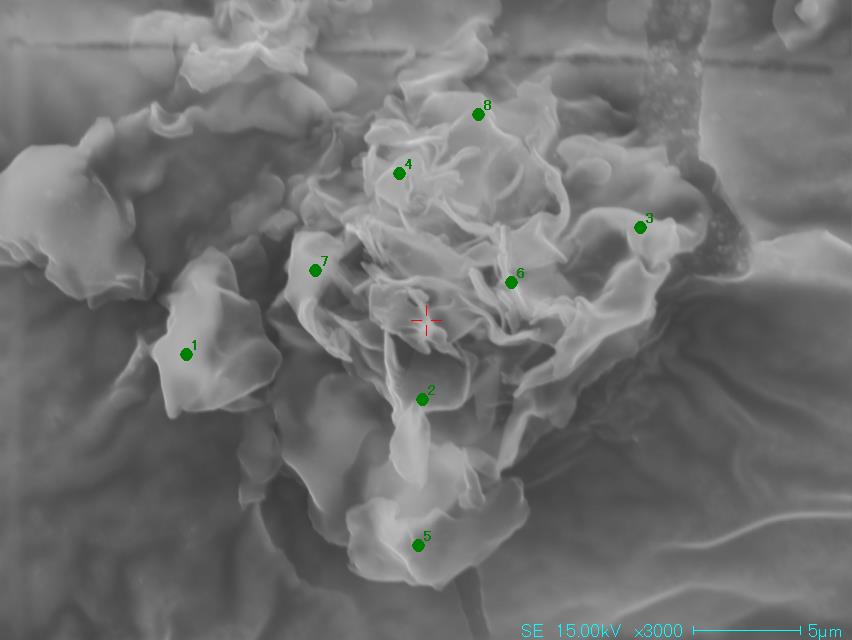


(Continued)


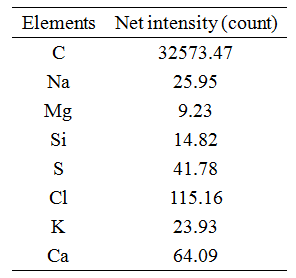

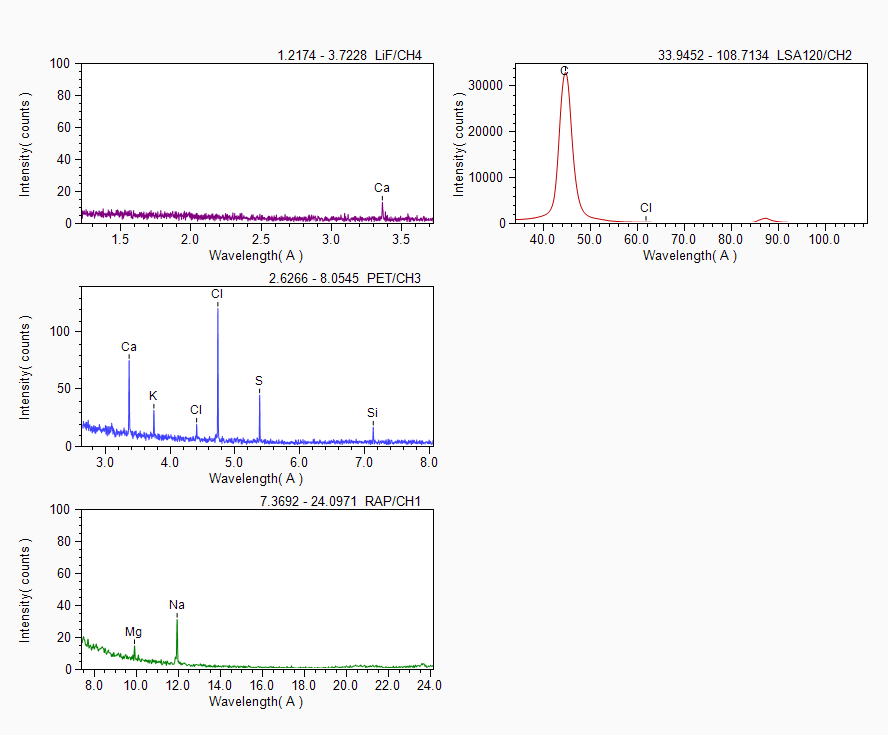


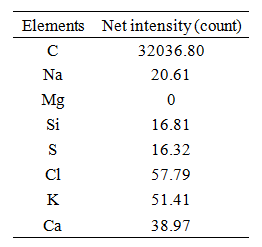

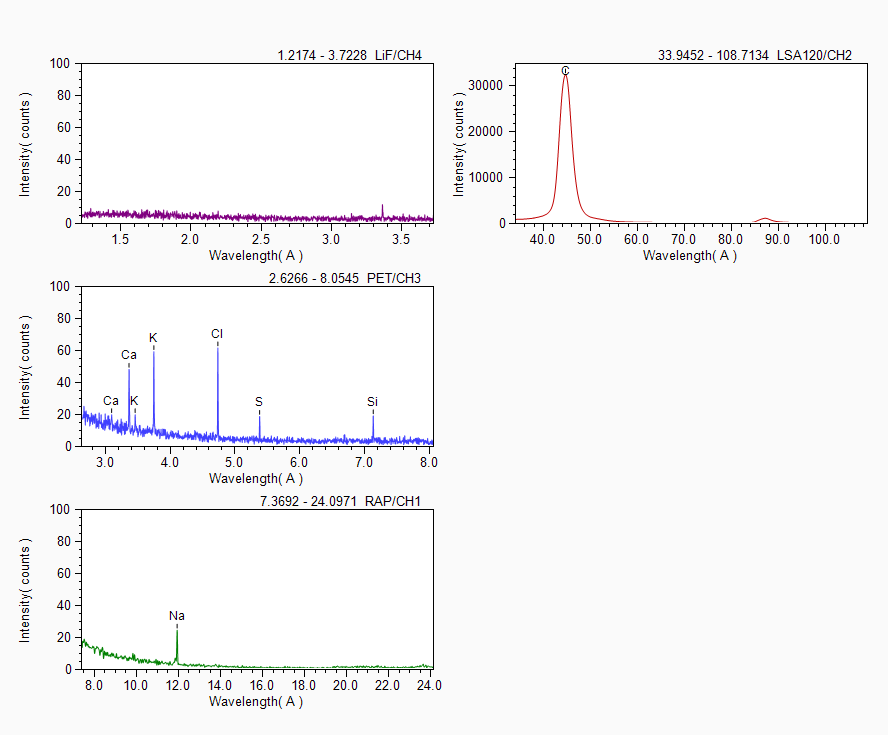


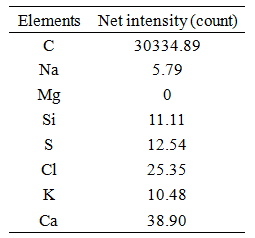

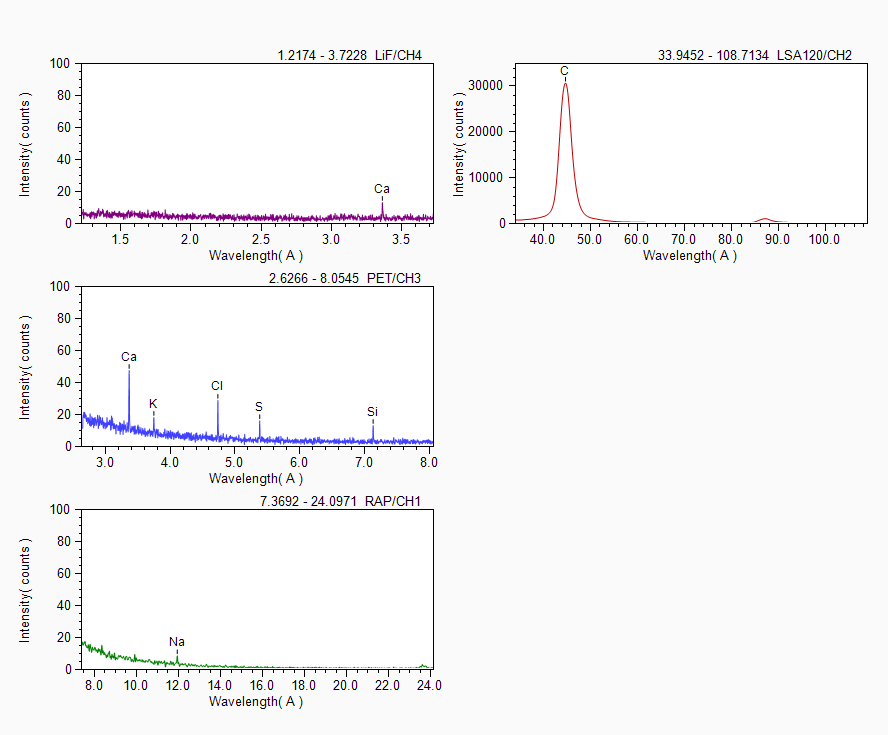


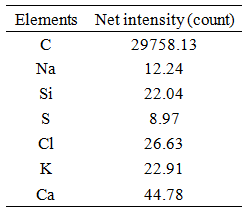

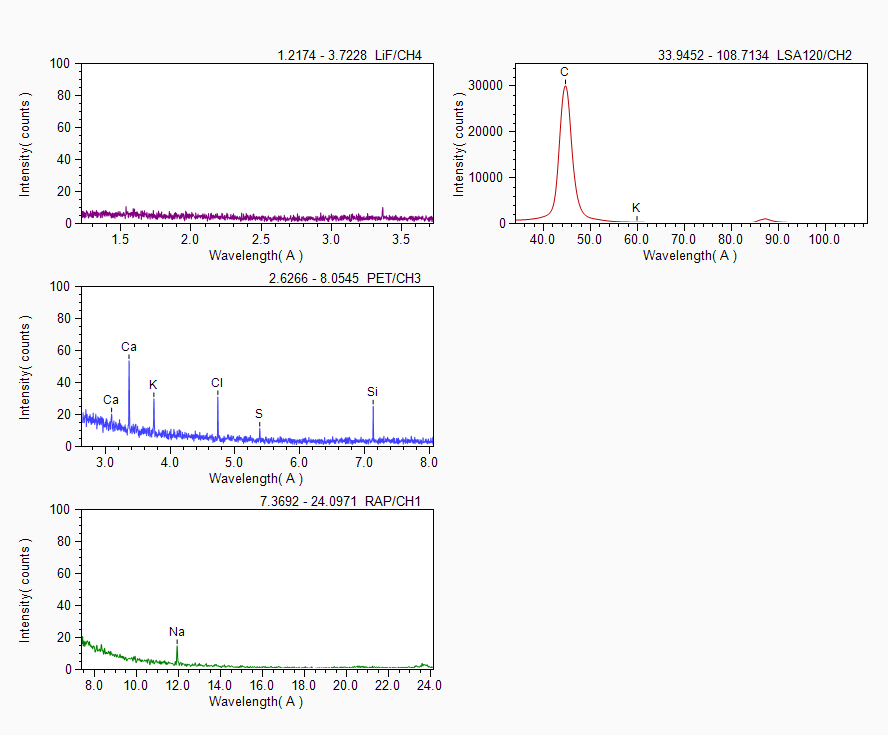


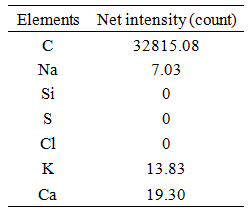

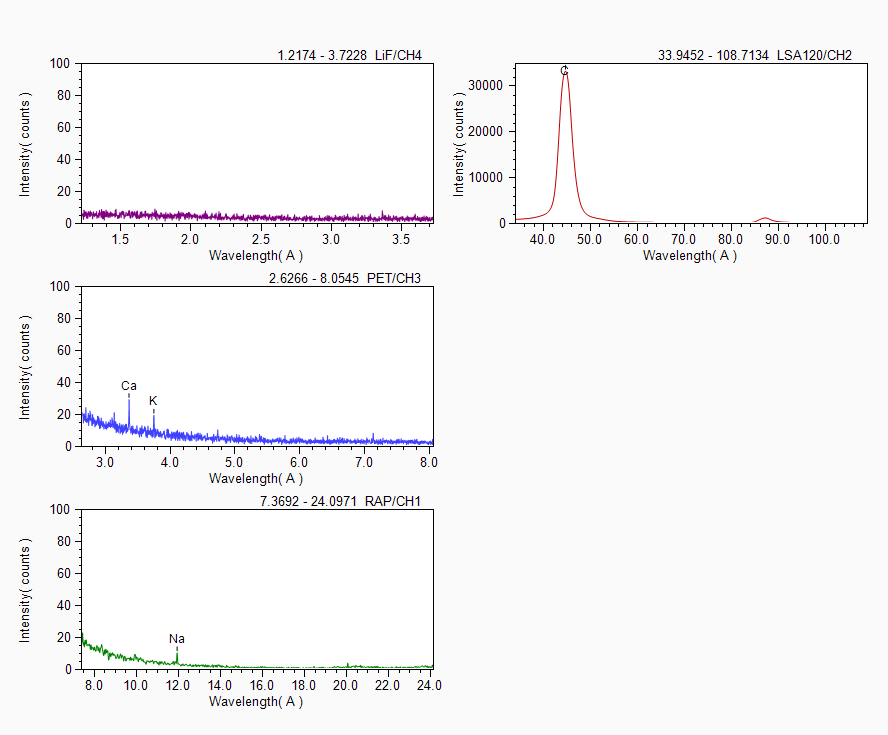


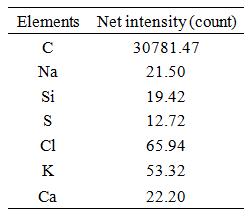

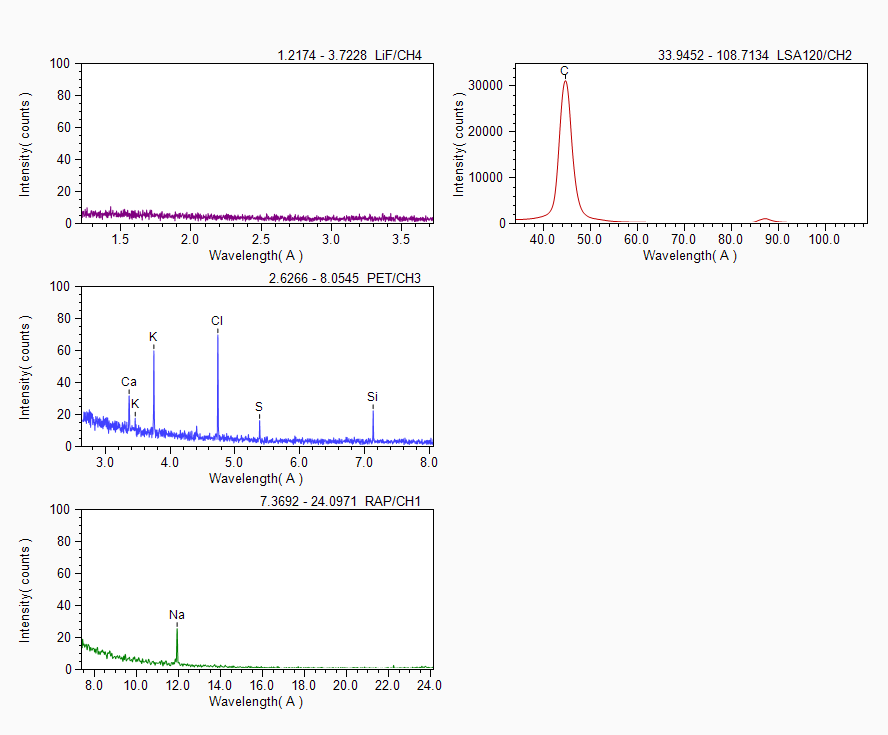


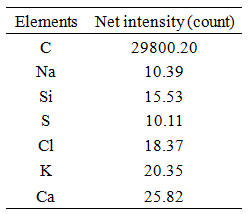

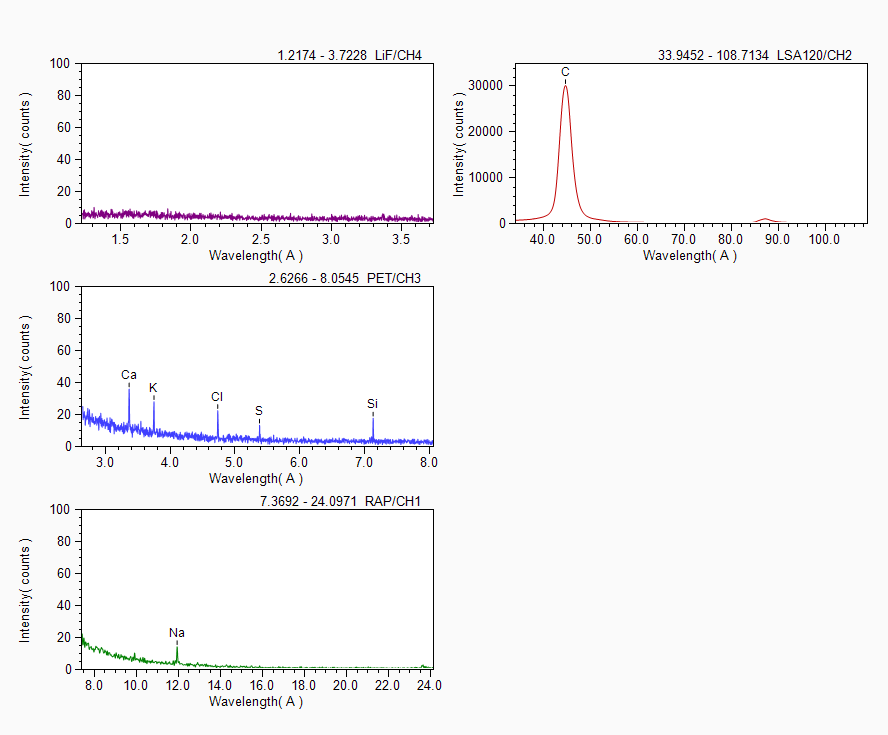


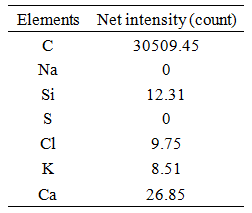

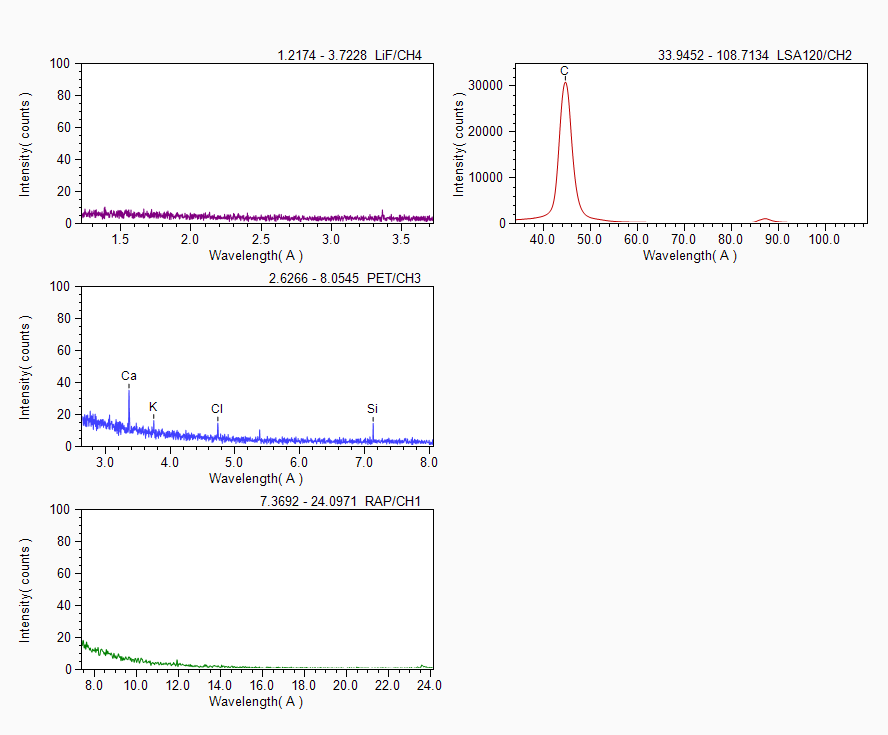


Figure S2. EPMA analysis of EF_PE_. (Image of sample area and the selected data points; the following figures are intensities for detecting elements for all the 8 data points.)
